# Widely Targeted Liver Metabolomics Reveals Potential Biomarkers in Mice with Drug-Induced Liver Injury

**DOI:** 10.3390/metabo16020096

**Published:** 2026-01-28

**Authors:** Jiangning Peng, Tingting Zhao, Xuehong Zhang, Hong Wang, Hui Li, Yan Liang

**Affiliations:** 1 College of Pharmacy, Hebei Medical University, Shijiazhuang 050017, China; 23034101844@stu.hebmu.edu.cn; 2 Hebei Institute of Drug and Medical Device Inspection, Shijiazhuang 050027, China; 1223056009@njupt.edu.cn; 3 School of Pharmacy, Henan University, Kaifeng 475004, China; 104753241330@henu.edu.cn; 4 McCombs Institute for the Early Detection and Treatment of Cancer, Houston, TX 77030, USA; 24034100571@stu.hebmu.edu.cn; 5 Hengshui Comprehensive Inspection and Testing Center, Hengshui 053000, China

**Keywords:** metabolomics, drug-induced liver injury, biomarkers, early diagnosis

## Abstract

Background: Drug-induced liver injury (DILI), a major type of adverse drug reaction, has become one of the leading causes of acute liver injury and liver failure worldwide. Its clinical significance lies not only in acute hepatocyte necrosis and functional failure but also in its role as a key initiating factor for liver cancer progression. Therefore, early diagnosis of DILI is of great importance. Methods: This study employed ultra-performance liquid chromatography-mass spectrometry (UPLC-MS/MS) to perform widely targeted metabolomics analysis on acetaminophen (APAP)-induced liver injury mice and healthy mice. Results: UPLC-QTRAP-MS/MS identified 41 differentially expressed metabolites primarily involved in glycerophospholipid metabolism, arginine and proline metabolism, primary bile acid biosynthesis, and glutathione metabolism pathways. The significant elevation of serum and hepatic alanine aminotransferase (ALT) and aspartate aminotransferase (AST) confirmed the successful establishment of the drug-induced liver injury (DILI) model. ROC curve analysis indicated 11 metabolites with AUC values exceeding 0.90 as potential biomarkers, including (R)-2-Hydroxybutyric acid, Glu-Gln, γ-Glu-Gln, 2-Methyllactic acid, L-Serine, Hyodeoxycholic acid, 3-Epideoxycholic acid, and Glycochenodeoxycholic acid 7-sulfate. Conclusions: We propose that these differential metabolites may serve as candidate biomarkers for DILI. Our findings provide a novel metabolomic signature derived directly from the injured tissue and offer a theoretical foundation for further research into early diagnosis of drug-induced liver injury.

## 1. Introduction

In recent years, global cancer incidence and mortality rates have been rapidly climbing, with liver cancer emerging as one of the most prevalent cancers [1]. As a critical early stage in liver cancer development, monitoring and screening for biomarkers of drug-induced liver injury (DILI) are of paramount importance [2].

The liver serves as the primary site for the metabolism and detoxification of drugs and exogenous substances. Drug-induced liver injury (DILI) results from damage to hepatocytes caused by drugs and their metabolites [3]. Acetaminophen (APAP), a widely used nonsteroidal anti-inflammatory drug (NSAID), leads to the formation of the toxic metabolite N-acetylbenzoquinoneimine (NAPQI) through oxidation by the CYP450 enzyme system. Excessive accumulation of NAPQI in the liver induces oxidative stress and mitochondrial dysfunction, triggering severe liver failure and progressively developing into hepatocellular carcinoma [4]. Acetaminophen-induced liver injury (AILI) has become a common cause of clinical liver disease.

The clinical diagnosis and monitoring of drug-induced liver injury (DILI) heavily rely on traditional serum biomarkers such as alanine aminotransferase (ALT), aspartate aminotransferase (AST), and total bilirubin (TBIL). These markers have significant limitations: while elevated levels indicate liver injury, they lack causative specificity, making it difficult to accurately distinguish DILI from other liver diseases [5]. There is an urgent clinical need to identify novel biomarkers beyond traditional indicators that offer greater specificity, sensitivity, and prognostic value. Developing and validating such markers is crucial for achieving personalized risk prediction, early intervention, improving long-term patient outcomes, and ensuring the safe clinical application of various drugs.

Metabolomics, a vital branch of systems biology, provides insights into disease mechanisms by conducting high-throughput analysis of endogenous metabolites in biological samples, directly reflecting the final state of an organism’s metabolic network. Abnormal fluctuations in the metabolome often precede the emergence of traditional clinical indicators [6]. This provides unique advantages to metabolomics in early disease diagnosis and clinical state identification. Therefore, this study employs broad-targeted metabolomics technology to analyze hepatic metabolic markers in mice with drug-induced liver injury, with the aim of identifying candidate DILI biomarkers and elucidating underlying metabolic disturbances. Direct profiling of the injury site can reveal mechanistic insights and prioritize metabolites for future validation in accessible biofluids like blood and urine [7,8]. This provides a theoretical foundation for the early clinical diagnosis of drug-induced liver injury.

## 2. Materials and Methods

### 2.1. Chemicals and Reagents

Chromatography-grade methanol and acetonitrile were purchased from Merck Drugs & Biotechnology (Merck, Darmstadt, Germany). Chromatography-grade acetic acid and ammonium formate were purchased from Beijing Dikma Technology Co., Ltd. (Beijing Dikma Technology Co., Ltd, Beijing, China); ammonia solution and formic acid were acquired from ThermoFisher Scientific Co., Ltd. (Thermo Fisher Scientific, Waltham, MA, USA). All were of chromatography grade. Chromatography columns were purchased from Waters(Waters, Shanghai, China). Acetaminophen was purchased from Shanghai Aladdin Biochemical Technology Co., Ltd (Shanghai, China). (Batch number: H21419345).

### 2.2. Establishment of the DILI Mice Model

Twenty 8-week-old male C57BL/6J mice (weighing 18–22 g) were randomly divided into two groups: a control group (*n* = 6) and an acetaminophen (APAP) model group (*n* = 6). The remaining mice were used in preliminary experiments to determine the appropriate APAP dose. The sample size was determined based on preliminary data and common practice in metabolomic studies to ensure statistical power [9]. After a one-week acclimation period, the mice were fasted for 12 h to deplete their glutathione levels. The control group received an equivalent volume of normal saline via intraperitoneal injection. The APAP model group was intraperitoneally injected with 300 mg/kg acetaminophen [10,11]. Nine hours after injection, the mice were euthanized by cervical dislocation, a time point previously shown to capture early metabolic changes before severe necrosis. Blood was collected for serum preparation, and liver tissues were immediately harvested: one portion was snap-frozen in liquid nitrogen for metabolomic analysis, and the other portion was fixed in 10% neutral buffered formalin for histology. This study was approved by the Ethics Committee of the Hebei Institute of Drug and Medical Device Inspection (No. LL2024-04) on 10 November 2024.

### 2.3. Serum, Liver Tissue Biochemistry and Histopathology

Serum and liver tissue ALT and AST activities were measured using commercial assay kits (Jiancheng Bioengineering Institute, Nanjing, China) according to the manufacturer’s instructions. Serum total bilirubin (TBIL) levels were also determined using a corresponding commercial assay kit (Jiancheng Bioengineering Institute, Nanjing, China) following the manufacturer’s protocol. For histopathology, after tissue dehydration, samples were embedded in paraffin, sectioned, and stained with hematoxylin and eosin (H&E). After mounting, pathological changes in mouse liver tissues were observed under a microscope (Leica, Wetzlar, Germany).

### 2.4. Sample Preparation and Extraction

The sample stored at −80 °C refrigerator was thawed on ice. The thawed sample was homogenized by a grinder (30 HZ) for 20 s. A 400 μL solution (Methanol: Water = 7:3, *V*/*V*) containing internal standard was added in to 20 mg grinded sample, and shaken at 2500 rpm for 5 min. After placing on ice for 15 min, the sample was centrifuged at 12,000 rpm for 10 min (4 °C). A 300 μL of supernatant was collected and placed in −20 °C for 30 min. The sample was then centrifuged at 12,000 rpm for 3 min (4 °C). A 200 μL aliquots of supernatant were transferred for LC-MS analysis. Quality control (QC) samples were prepared by blending extracts from the above samples. During instrumental analysis, one QC sample was inserted for every ten analytical samples to monitor the repeatability of the analytical process. The details of the stability and reliability Analysis of Detection Method was shown in Supplementary Materials.

### 2.5. UPLC Conditions

The sample extracts were analyzed using an LC-ESI-MS/MS system (Sciex, Framingham, MA, USA). The analytical conditions were as follows, UPLC: column, Waters ACQUITY UPLC HSS T3 C18 (1.8 µm, 2.1 mm × 100 mm); column temperature, 40 °C; flow rate, 0.4 mL/min; injection volume, 2 μL; solvent system, water (0.1% formic acid): acetonitrile (0.1% formic acid); solvent B gradient program, 5% to 20% in 2 min, increased to 60% in the following 3 min, increased to 99% in 1 min and held for 1.5 min, then come back to 5% within 0.1 min, held for 2.4 min.

### 2.6. MS Conditions

LIT and triple quadrupole (QQQ) scans were acquired on a triple quadrupole-linear ion trap mass spectrometer (QTRAP), QTRAP LC-MS/MS System, equipped with an ESI Turbo Ion-Spray interface, operating in positive and negative ion mode and controlled by Analyst 1.6.3 software (Sciex). The ESI source operation parameters were as follows: source temperature 500 °C; ion spray voltage (IS) 5500 V (positive), −4500 V (negative); ion source gas I (GSI), gas II (GSII), curtain gas (CUR) were set at 55, 60, and 25.0 psi, respectively; the collision gas (CAD) was high. Instrument tuning and mass calibration were performed with 10 and 100 μmol/L polypropylene glycol solutions in QQQ and LIT modes, respectively. A specific set of MRM transitions was monitored for each period according to the metabolites eluted within this period.

### 2.7. Data Processing and Statistical Analysis

Unsupervised PCA was performed using the prcomp function in R. For two-group comparisons, differential metabolites were identified using a combination of VIP > 1.0 from the OPLS-DA model and *p*-value < 0.05 from Student’s *t*-test. The OPLS-DA and permutation tests (200 permutations) were conducted using the MetaboAnalystR package in R(4.2.0). All metabolomics data were log2-transformed and mean-centered prior to multivariate analysis. For serum biochemistry data, results are presented as mean ± standard deviation (SD). Differences between the two groups were analyzed using Student’s *t*-test. A *p*-value < 0.05 was considered statistically significant. Metabolite identification and pathway mapping were performed using the KEGG database.

## 3. Results

### 3.1. Validation of the APAP-Induced DILI Model

**Figure 1:**
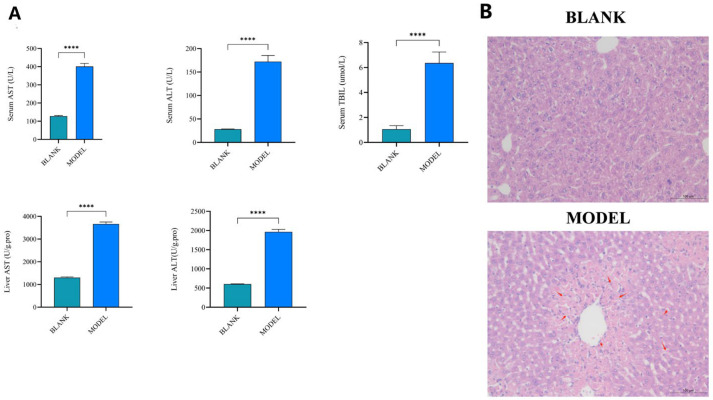
Validation of drug-induced liver injury (DILI) in mice. (**A**) Serum and liver tissue levels of ALT and AST in control and acetaminophen (APAP)-treated mice; Serum level of TBIL. Data are presented as mean ± standard deviation (*n* = 6). **** *p* < 0.001 vs. control group (Student’s *t*-test). (**B**) Representative hematoxylin and eosin (H&E) stained liver tissue sections from control and model groups. Scale bar = 100 μm. Red arrows indicate areas of necrosis.

To confirm the establishment of liver injury, we assessed serum and hepatic biochemical indices as well as liver histopathology. Compared with the control group, the acetaminophen-treated group showed significantly elevated serum and hepatic levels of alanine aminotransferase (ALT), aspartate aminotransferase (AST) and total bilirubin (TBIL), (*p* < 0.001; Figure 1A). Histopathological examination revealed characteristic centrilobular necrosis and inflammatory cell infiltration in the livers of APAP-treated mice, which were absent in the control group (Figure 1B). These results confirm the successful induction of drug-induced liver injury (DILI), providing a foundation for subsequent metabolomic analysis.

### 3.2. Stability and Reliability Analysis of Detection Method

Pearson correlation analysis was performed on the QC samples. Higher correlation among QC samples (|r| approaches 1) indicates greater stability throughout the testing process and higher data quality. As shown in Figure S1, the correlations among QC samples all exceed 0.990 and progressively approach 1.

The CV value, or Coefficient of Variation, is the ratio of the standard deviation of raw data to the mean of the raw data, reflecting the degree of data dispersion. Using the Empirical Cumulative Distribution Function (ECDF), one can analyze the frequency of substances with CV values below a reference threshold. A higher proportion of substances with lower CV values in QC samples indicates greater experimental data stability. In Figure S2, over 75% of the substances in the QC sample exhibit a CV value below 0.3, indicating highly stable experimental data.

Principal component analysis (PCA) was performed on all sample groups and QC samples to assess overall metabolite differences between groups and the degree of variation within samples. Figure S3 shows that the data for each sample group are tightly clustered, indicating that the detection method and samples exhibit good stability and reproducibility.

### 3.3. Stability and Applicability Analysis of DILI Model

**Figure 2:**
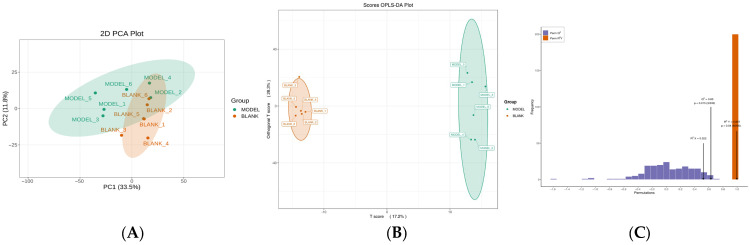
Stability and applicability analysis of the DILI model. (**A**) PCA plot of the disease group and the healthy control group. (**B**) OPLS-DA Scores [1]. (**C**) OPLS-DA scores and OPLS-DA replacement tests for Model vs. Blank. The values represented by the black arrows correspond to the R^2^Y and Q^2^ values of the original model.

Principal component analysis (PCA) and OPLS-DA were performed on metabolites detected by UPLC-QTRAP-MS to examine metabolic variability between diseased and healthy mice. The PCA score plot revealed tight clustering of samples within each group (Figure 2A), indicating excellent stability and reproducibility of the data.

Model quality was assessed using the goodness-of-fit parameter R^2^Y and the cross-validation capability parameter Q^2^Y. As shown in Figure 2B, the OPLS-DA score plot clearly revealed a distinct separation trend between the experimental and control groups, indicating significant differences in metabolic phenotypes between the two sample categories. The model exhibited an R^2^Y value of 0.998, approaching 1.0, demonstrating strong interpretive power for the data; the Q^2^Y value was 0.639 (Figure 2C), indicating good predictive capability. To test for model overfitting, a permutation test was conducted. Results showed that the permutation-adjusted R^2^ intercept and Q^2^ intercept on the left were both lower than the original values on the right, and the Q^2^ intercept was less than 0, meeting the model validity criteria. This confirms the metabolic model is stable and reliable, with no evidence of overfitting.

### 3.4. Widely Targeted Metabolomics Analysis of DILI Mice

**Figure 3:**
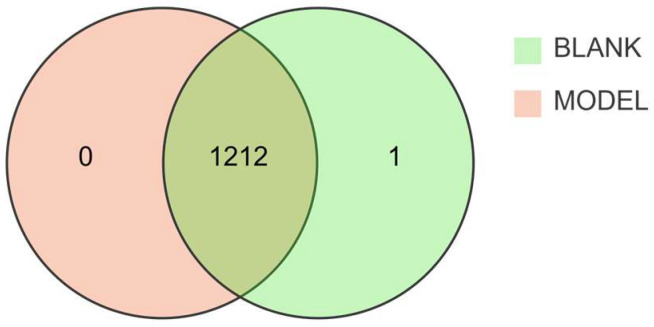
Venn diagram of metabolites. The green section represents metabolites from the healthy control group, while the orange section represents metabolites from the disease group.

Experimental results revealed that 1213 metabolites were detected using UPLC-QTRAP-MS. The Venn diagram (Figure 3) indicated 1212 differential metabolites between the healthy group and the model groups.

Differential metabolite analysis was performed on the detected metabolites, screening for differences based on VIP values, significance *p*-values, and FC (Fold change). The screening criteria were applied sequentially as follows: (1) *p*-value < 0.05; (2) VIP value > 1.00; (3) FC value > 1 or <0.90. This study identified 41 significantly different metabolites (Table 1), with their mass spectrometry data presented in Table S1. Compared to the healthy group, the diseased group exhibited significantly elevated levels of metabolites including 2-Methyllactic acid, (R)-2-Hydroxybutyric acid, Glu-Gln, γ-Glu-Gln, 4-Methoxyestrone, 2-Methoxyestrone, and Acetylcholine. while the concentrations of Cholic acid, L-Serine, Tauroursodeoxycholic acid, Hyodeoxycholic acid, 2,2-Dimethylglutaric acid, and Glycochenodeoxycholic acid 7-sulfate were significantly downregulated.

**Figure 4:**
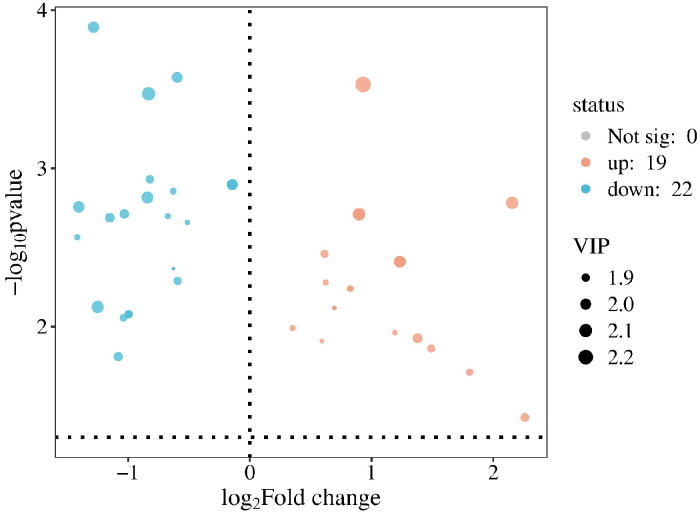
Volcano plot of metabolites differing between disease groups and healthy controls. Blue dots represent significantly downregulated metabolites; red dots represent significantly upregulated metabolites. The black dots separate the differential metabolites into two regions: downregulated metabolites on the left and upregulated metabolites on the right.

Based on the screened differential metabolites, a volcano plot (Figure 4) was generated to visualize the relative abundance differences in metabolites between the disease and healthy groups, as well as their statistical significance. The plot uses log2 (Fold Change) and −log10 (*p*-value) as the X and Y axes, respectively. The distribution of significantly altered metabolites in the volcano plot shows that 19 metabolites were significantly upregulated, while 22 metabolites were significantly downregulated in the model group compared to the healthy control group.

**Figure 5:**
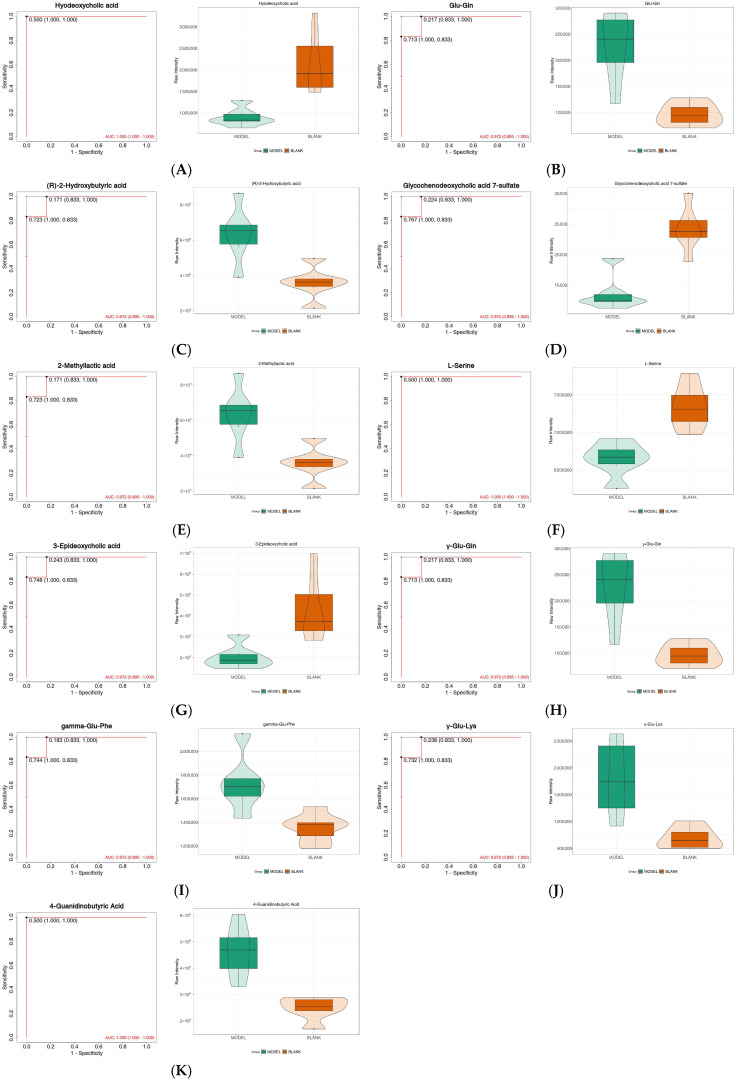
ROC curves and violin plots for the top 11 candidate biomarkers. (**A**–**K**) (**Left**): ROC curves for each metabolite. (**Right**): Corresponding violin plots showing metabolite distribution in Blank group and DILI groups.

Area under the ROC curve (AUC) analysis was employed to assign clinical diagnostic value to the differential metabolites identified between the model and healthy groups. Metabolites with an AUC > 0.90 were considered potential biomarkers. The results (Figure 5) showed that, among the 41 differential metabolites, 11 biomarkers including Glycochenodeoxycholic acid 7-sulfate, Hyodeoxycholic acid, Glu-Gln, γ-Glu-Gln, and 2-Methyllactic acid exhibited good AUC values. The distribution and probability density of these potential biomarkers were visualized using violin plots. The analysis revealed that the levels of biomarkers such as (R)-2-Hydroxybutyric acid, Glu-Gln, γ-Glu-Gln, and 2-Methyllactic acid were significantly increased in the disease group, while the levels of biomarkers including L-Serine, Hyodeoxycholic acid, 3-Epideoxycholic acid, and Glycochenodeoxycholic acid 7-sulfate were significantly decreased in the disease group.

### 3.5. Enrichment Analysis of Metabolic Pathways for Differential Markers

**Figure 6:**
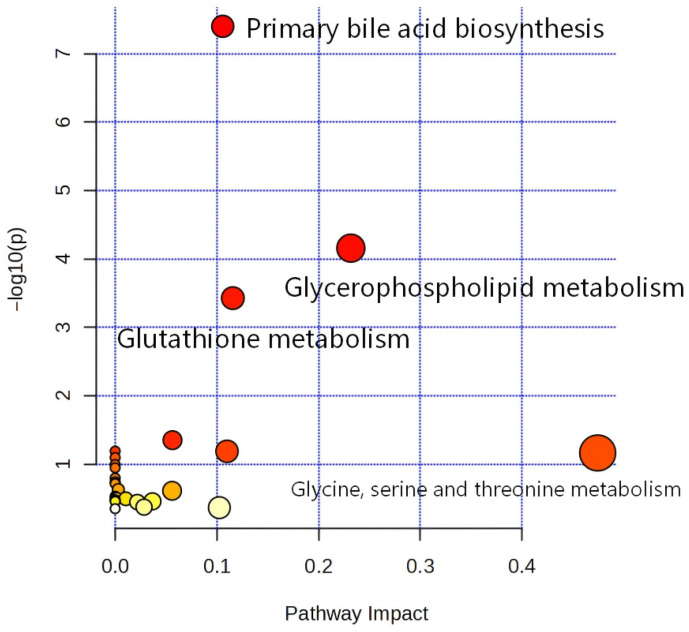
Enrichment Analysis of Metabolic Pathways. The darker the dot color, the higher the enrichment level of metabolites in that metabolic pathway and the greater the statistical significance.

To investigate changes in metabolic pathways during the disease progression of DILI mice, a metabolic pathway enrichment analysis was performed on 41 differentially expressed metabolites using MetaboAnalyst 6.0 (Figure 6). Metabolic pathways with *p*-values < 0.05 were considered to have a significant impact on metabolites (Table 2). Metabolic pathways including primary bile acid biosynthesis, glutathione metabolism, and glycerophospholipid metabolism exerted regulatory effects on DILI, significantly influencing metabolite alterations.

## 4. Discussion

With the increasing prevalence and progressive deterioration of hepatocellular carcinoma, drug-induced liver injury (DILI)—a critical pathogenic stage in disease progression—urgently requires clinical management and early diagnostic strategies. In this study, widely targeted metabolomics was applied to identify differential metabolites between diseased and healthy mice. By integrating differential metabolite screening with ROC analysis, 11 biomarkers with promising clinical application potential were identified (*p* < 0.05, AUC > 0.90), including Glycochenodeoxycholic acid 7-sulfate, Hyodeoxycholic acid, Glu-Gln, γ-Glu-Gln, 2-Methyllactic acid, γ-Glu-Lys, 3-Epideoxycholic acid, (R)-2-Hydroxybutyric acid, γ-Glu-Phe, L-Serine, and 4-Guanidinobutyric acid. KEGG pathway analysis revealed that, compared with the healthy group, differential metabolites in the disease group were primarily enriched in metabolic pathways such as primary bile acid biosynthesis, glutathione metabolism, and glycerophospholipid metabolism.

Before the metabolomic analysis, we employed a single acetaminophen dosing regimen of 300 mg/kg to simulate sustained metabolic stress and early-stage acute liver injury, rather than using high doses such as 600–1000 mg/kg to induce acute liver failure [12]. This model yields consistent and reproducible metabolic alterations with low mortality, making it suitable for biomarker discovery research focused on the progression of injury [9,10,11]. Successful establishment of the drug-induced liver injury mouse model was confirmed by serum and hepatic biochemical results. Histopathological examination further revealed marked histopathological alterations in the disease group.

The experiment revealed that the occurrence of DILI is closely associated with dysregulation of the primary bile acid biosynthesis pathway [13]. Differential metabolites involved in primary bile acid metabolism were significantly downregulated in the DILI group, indicating that APAP severely impairs hepatic bile acid synthesis and metabolism. The decreased level of cholic acid, a primary bile acid, in the disease group reflects suppressed activity of cholesterol 7α-hydroxylase (CYP7A1) in hepatocytes. Bile acids can inhibit the de novo synthesis of bile acids by activating the farnesoid X receptor (FXR) and downstream signaling pathways, such as the JNK/c-Jun pathway, which in turn downregulates CYP7A1 expression via negative feedback [14]. Under DILI conditions, hepatocellular damage and inflammatory responses exacerbate this regulatory mechanism, leading to reduced synthesis of cholic acid and its derivatives—a finding consistent with our observations. γ-muricholic acid, a major primary bile acid, plays a role in modulating FXR signaling and suppressing hepatic lipid accumulation and inflammation [15]. Its decline weakens the protective effect against lipid peroxidation and inflammatory responses in hepatocytes, thereby aggravating liver injury. As a secondary bile acid, the reduced level of hyodeoxycholic acid alters the composition of the bile acid pool and the efficiency of enterohepatic circulation, resulting in disordered lipid metabolism and accumulation of toxic bile acids [16]. Reported evidence suggests that hyodeoxycholic acid, produced by gut microbiota metabolism, possesses hepatocyte-repairing and antioxidative stress capabilities. Its marked decrease during DILI progression diminishes the body’s capacity to counteract oxidative stress, thereby worsening liver injury—a result aligned with our findings [17]. Notably, hyodeoxycholic acid demonstrated a favorable AUC value (>0.90) in this study, supporting its potential as an early diagnostic biomarker in clinical practice. Glycochenodeoxycholic acid 7-sulfate, a key product of bile acid sulfation metabolism, decreased in our model, indicating impaired sulfotransferase activity or obstruction of the bile acid sulfation pathway in hepatocytes [18,19]. Sulfation is a critical step in bile acid detoxification and excretion; its disruption leads to intrahepatic accumulation of unconjugated bile acids, further inducing hepatocyte apoptosis and cholestasis [20]. In summary, the significant downregulation of bile acid metabolites during DILI progression may collectively reflect the pathological state of impaired hepatocyte synthetic function and disrupted metabolic pathways. Dynamic monitoring of these bile acid metabolites, especially when combined into a characteristic “Bile acid signature” demonstrates potential as highly specific candidate biomarkers for the early diagnosis and progression monitoring of DILI.

The occurrence of DILI originates from the overdose of drugs such as APAP, whose toxic metabolites lead to the massive generation of reactive oxygen species (ROS), triggering systemic oxidative stress and subsequently causing severe disruption of hepatic GSH metabolic homeostasis. The imbalance in GSH homeostasis directly alters the redox state of hepatocytes and affects downstream signal transduction pathways [21]. Our KEGG pathway enrichment analysis showed that differential metabolites were significantly enriched in the glutathione metabolism pathway. Among them, the levels of γ-glutamyl dipeptides such as γ-glutamylglutamine (γ-Glu-Gln), γ-glutamyllysine (γ-Glu-Lys), and γ-glutamylphenylalanine (γ-Glu-Phe) were significantly upregulated. The γ-glutamyl cycle is a central metabolic circuit in the body for GSH synthesis, degradation, and amino acid transmembrane transport. The accumulation of these dipeptides, as key intermediate metabolites of this cycle, directly reflects a compensatory sharp increase in γ-glutamyl cycle flux under oxidative stress, aimed at accelerating GSH regeneration and synthesis to counteract ROS assault [22]. Consequently, the upregulation of γ-Glu-Gln and γ-Glu-Lys may serve as sensitive metabolic markers in the early stage of DILI, indicating that cellular defense mechanisms remain in an effective compensatory phase. The elevation of these dipeptides acts as a sensitive indicator of sustained oxidative stress, potentially occurring prior to hepatocyte necrosis, and their correlation with systemic markers deserves further investigation in the future.

The results showed that, compared with the healthy group, differential metabolites such as LPE (0:0/20:4), LPC (16:2/0:0), and LPC (0:0/16:2) were enriched in the glycerophospholipid metabolism pathway, and their levels were significantly downregulated in the DILI group. The liver is a central organ for lipid metabolism. Glycerophospholipids are the main components of the cell membrane bilayer, and their homeostasis is crucial for maintaining membrane structure, fluidity, and function [23]. This study observed a decrease in the levels of various lysophospholipids, which may reveal deeper metabolic disturbances in APAP-induced acute liver injury. The decline in these lysophospholipid levels is more likely due to excessive consumption or impaired synthesis of their precursor membrane phospholipids, such as phosphatidylcholine and phosphatidylethanolamine [24]. Under drug toxicity, the massive production of reactive oxygen species attacks cell membranes, leading to extensive degradation of membrane phospholipids. Simultaneously, severe liver injury may impair the de novo synthesis capacity of phospholipids [25,26]. Therefore, the decrease in lysophospholipids is not a simply beneficial change but rather a metabolic marker indicating that the hepatocyte membrane system is under severe attack and that the balance between synthesis and degradation has been disrupted [27,28]. This disruption of membrane phospholipid metabolism entails dual detrimental effects. First, it directly impairs the integrity and function of hepatocyte membranes and organelle membranes, leading to hepatocyte swelling, rupture, and even apoptosis [29]. Second, by affecting the function of membrane-bound organelles such as the endoplasmic reticulum, it may indirectly interfere with the normal operation of drug-metabolizing enzyme systems like the CYP450 enzyme system that depend on these structures, thereby creating a vicious cycle that exacerbates drug accumulation and liver injury [30,31]. Lysophospholipids such as LPC themselves also serve as important lipid messengers, and disruption of their homeostasis may further perturb intracellular signaling [32,33,34]. Therefore, the downregulation of specific lysophospholipids such as LPE (0:0/20:4) and LPC (16:2/0:0) can serve as sensitive indicators reflecting hepatocyte membrane damage and disruption of lipid metabolism homeostasis. We propose that combined detection of these specific lipid metabolites with traditional liver enzyme indicators will facilitate earlier and more comprehensive assessment of the severity and prognosis of drug-induced liver injury.

This study provides new evidence for metabolomic research on DILI caused by APAP overdose. Although previous studies have identified candidate biomarkers in plasma or serum [35,36], our direct analysis of liver tissue offers a unique perspective on local metabolic disturbances at the site of injury. This approach can reveal metabolites that are more concentrated or specific to hepatic pathology, thereby informing the selection of targets for future blood-based assay development.

## 5. Conclusions

We identified 41 differential metabolites in the livers of DILI mice using widely targeted metabolomics. Critically, the DILI model was validated by significant increases in ALT/AST of serum and tissue and characteristic histopathological damage. Among these, 11 metabolites, including Glycochenodeoxycholic acid 7-sulfate, Hyodeoxycholic acid, Glu-Gln, γ-Glu-Gln, and 2-Methyllactic acid, showed high diagnostic potential (AUC > 0.90) as candidate biomarkers. Pathway analysis implicated primary bile acid biosynthesis, glutathione metabolism, and glycerophospholipid metabolism in DILI pathogenesis. While this study provides a direct view of hepatic metabolic changes, future work should focus on translating these liver-derived candidate biomarkers into clinically accessible biofluids like plasma and urine and validating them in larger cohorts and different etiologies of DILI.

## Figures and Tables

**Table 1:** Differentially expressed metabolites identified by widely targeted metabolomics.

Compounds	Type	Log2FC	VIP	*p*-Value
2-Methyllactic acid	up	0.825465845	1.861342901	0.005760072
(R)-2-Hydroxybutyric acid	up	0.825465845	1.861342901	0.005760072
Glu-Gln	up	1.23222829	2.033086617	0.003888383
γ-Glu-Gln	up	1.23222829	2.033086617	0.003888383
4-Methoxyestrone	up	0.693202533	1.833980564	0.007628821
2-Methoxyestrone	up	0.693202533	1.833980564	0.007628821
Acetylcholine	up	0.897419019	2.070768801	0.001950573
4-Guanidinobutyric acid	up	0.897419019	2.070768801	0.001950573
D-Glucosamine 6-Phosphate	up	0.624203269	1.852145893	0.005262299
Putrescine	up	2.259126796	1.921946776	0.037564691
Carnitine C20:2	up	0.613922485	1.898151526	0.003466492
3-Carboxypropyltrimethylammonium	up	0.930645718	2.266682683	0.000294884
n-Oleoylethanolamine	up	1.806254314	1.877942884	0.019437404
Carnitine C8:0	up	1.490148539	1.897794513	0.013758121
N-Methylisoleucine	up	2.153820406	2.06886256	0.001651802
γ-Glu-Lys	up	1.378132354	1.954322115	0.011864334
γ-Glu-Phe	up	0.351038603	1.854460271	0.010220471
LPC (0:0/16:2)	up	0.592482236	1.836169766	0.012357526
Carnitine C6-OH	up	1.191688825	1.843090325	0.010925769
Indole-4-carboxaldehyde	down	−0.819874057	1.909557306	0.001171704
Mucic acid	down	−0.62711227	1.82915026	0.004303106
Glucaric acid	down	−0.62711227	1.82915026	0.004303106
Cholic acid	down	−0.994387372	1.896668974	0.008358778
γ-muricholic acid	down	−0.994387372	1.896668974	0.008358778
L-Serine	down	−0.143082272	2.000175592	0.001265731
L-Isoserine	down	−0.143082272	2.000175592	0.001265731
Tauroursodeoxycholic acid	down	−1.404456722	2.019322706	0.001750595
Hyodeoxycholic acid	down	−1.249398986	2.05621151	0.007516161
13-HOTrE	down	−1.037089103	1.88732953	0.00879502
9(S)-HpOTrE	down	−0.841196781	2.038868729	0.001526687
3-Epideoxycholic acid	down	−1.080181454	1.92808492	0.015495934
Ribonic acid	down	−0.628392013	1.86566919	0.001392447
2,2-Dimethylglutaric acid	down	−1.14953439	1.945250245	0.002052736
Glycochenodeoxycholic acid 7-sulfate	down	−0.830802263	2.122140314	0.000337779
9-deoxy-9-methylene-PGE2	down	−1.416794101	1.854311763	0.002720152
Indole-3-Carboxaldehyde	down	−0.674207969	1.851828401	0.002009603
LPC (17:2/0:0)	down	−0.511700545	1.843496623	0.002197334
LPC (0:0/18:3)	down	−1.030378647	1.942115118	0.00194038
1-Aminopentadecane	down	−0.596160213	1.991094633	0.00026619
LPC (18:3/0:0)	down	−0.592590883	1.905318155	0.005142233
3-Aminophenol	down	−1.28232858	2.016573337	0.00012809

**Table 2:** Metabolite Pathway Enrichment Analysis Table.

	Total	Hits	*p* Value	Impact
Primary bile acid biosynthesis	46	8	3.9883 × 10^−8^	0.10576
Glycerophospholipid metabolism	36	5	0.00006966	0.23171
Glutathione metabolism	28	4	0.00037373	0.11571
One carbon pool by folate	26	2	0.044534	0.05637
Linoleic acid metabolism	5	1	0.064316	0
Glyoxylate and dicarboxylate metabolism	32	2	0.064829	0.11
Glycine, serine and threonine metabolism	33	2	0.068471	0.4744
Arginine and proline metabolism	36	2	0.079797	0
Taurine and hypotaurine metabolism	8	1	0.10099	0
Ascorbate and aldarate metabolism	9	1	0.11291	0
alpha-Linolenic acid metabolism	13	1	0.1591	0
D-Amino acid metabolism	15	1	0.18132	0
Glycerolipid metabolism	16	1	0.19223	0
Fructose and mannose metabolism	20	1	0.23447	0.00311
beta-Alanine metabolism	21	1	0.24469	0.05597
Glycolysis or Gluconeogenesis	26	1	0.29391	0
Alanine, aspartate and glutamate metabolism	28	1	0.31272	0
Lipoic acid metabolism	28	1	0.31272	0.0017
Steroid hormone biosynthesis	87	2	0.32004	0.0104
Inositol phosphate metabolism	30	1	0.33106	0
Porphyrin metabolism	31	1	0.34005	0
Sphingolipid metabolism	32	1	0.34893	0
Glycosylphosphatidylinositol (GPI)-anchor biosynthesis	32	1	0.34893	0.03665
Cysteine and methionine metabolism	33	1	0.3577	0.02184
Steroid biosynthesis	41	1	0.42388	0.02837
Amino sugar and nucleotide sugar metabolism	42	1	0.43168	0.10245
Arachidonic acid metabolism	44	1	0.44698	0

## Data Availability

The data that support the findings of this study are available from the corresponding author, upon reasonable request.

## Supplementary Materials

The following supporting information can be downloaded at: https://www.mdpi.com/article/10.3390/metabo16020096/s1, Figure S1: QC Sample Correlation Plot. The diagonal squares represent QC sample names; Figure S2: Empirical cumulative distribution function plot of the coefficient of variation (CV) values for QC samples; Figure S3: Principal Component Analysis Plot of Experimental Group Samples and QC Samples; Table S1: Mass spectrometric characterization of metabolites.

## Abbreviations

The following abbreviations are used in this manuscript:

AILI	Acetaminophen-induced liver injury
ALT	Alanine aminotransferase
APAP	Acetaminophen
AST	Aspartate aminotransferase
AUC	Area under the ROC curve
CAD	Collision gas
CV	Coefficient of variation
CUR	Curtain gas
CYP7A1	Cholesterol 7α-hydroxylase
DILI	Drug-induced liver injury
ECDF	Empirical cumulative distribution function
FC	Fold change
FXR	Farnesoid X receptor
GSI	Ion source gas I
GSII	Ion source gas II
GSH	Glutathione
IS	Ion spray voltage
JNK	c-Jun N-terminal kinase
LPC	Lysophosphatidylcholine
LPE	Lysophosphatidylethanolamine
LIT	Linear ion trap
MRM	Multiple reaction monitoring
NAPQI	N-acetylbenzoquinoneimine
NSAID	Nonsteroidal anti-inflammatory drug
OPLS-DA	Orthogonal partial least squares-discriminant analysis
PCA	Principal component analysis
QC	Quality control
QQQ	Triple quadrupole
QTRAP	Triple quadrupole-linear ion trap mass spectrometer
ROC	Receiver operating characteristic
ROS	Reactive oxygen species
TBIL	Total bilirubin
UPLC-MS/MS	Ultra-performance liquid chromatography-tandem mass spectrometry
VIP	Variable importance in projection

## References

[1] Germani G., Battistella S., Ulinici D., Zanetto A., Shalaby S., Pellone M., Gambato M., Senzolo M., Russo F.P., Burra P. (2021). Drug induced liver injury: From pathogenesis to liver transplantation. Minerva Gastroenterol..

[2] Li X., Tang J., Mao Y. (2022). Incidence and risk factors of drug-induced liver injury. Liver Int..

[3] Zhou Y., Wang J., Zhang D., Liu J., Wu Q., Chen J., Tan P., Xing B., Han Y., Zhang P. (2021). Mechanism of drug-induced liver injury and hepatoprotective effects of natural drugs. Chin. Med..

[4] Ozturk N.B., Uskudar E., Toruner M.D., Simsek C., Gurakar A. (2025). Drug-induced liver injury: Diagnosis, management and the role of liver transplantation. Hepatol. Forum.

[5] Buzatto A.Z., de Sousa A.C., Guedes S.F., Cieslarová Z., Simionato A.V.C. (2014). Metabolomic investigation of human diseases biomarkers by CE and LC coupled to MS. Electrophoresis.

[6] Bu F., Shen X., Zhan H., Wang D., Min L., Song Y., Wang S. (2025). Efficient Metabolomics Profiling from Plasma Extracellular Vesicles Enables Accurate Diagnosis of Early Gastric Cancer. J. Am. Chem. Soc..

[7] Zhu Y., Fan Y., Chen L., Shang Y., Feng Z., Fan L., Ouyang H., He J. (2025). Protective effect and mechanism of *Perilla frutescens* (L.) Britton on acute liver injury via an integrated strategy of pharmacodynamics, liver metabonomics and lipidomics, and network pharmacology. J. Funct. Foods.

[8] Nikopoulou C., Kleinenkuhnen N., Parekh S., Sandoval T., Ziegenhain C., Schneider F., Giavalisco P., Donahue K.-F., Vesting A.J., Kirchner M. (2023). Spatial and single-cell profiling of the metabolome, transcriptome and epigenome of the aging mouse liver. Nat. Aging.

[9] Wang Z., Zhao M.-Y., Wang J.-W., He Y.-T., Yang R.-Q., Ji J.-Q., Ding Y., Wang C., Zhao Y.-H., Zhang W.-K. (2025). The GSK3β inhibitor attenuate APAP-induced liver injury by regulating PGC1α-mediated oxidative stress. Int. Immunopharmacol..

[10] Nguyen N.T., Umbaugh D.S., Smith S., Adelusi O.B., Sanchez-Guerrero G., Ramachandran A., Jaeschke H. (2023). Dose-dependent pleiotropic role of neutrophils during acetaminophen-induced liver injury in male and female mice. Arch. Toxicol..

[11] Bhushan B., Walesky C., Manley M., Gallagher T., Borude P., Edwards G., Monga S.P., Apte U. (2014). Pro-Regenerative Signaling after Acetaminophen-Induced Acute Liver Injury in Mice Identified Using a Novel Incremental Dose Model. Am. J. Pathol..

[12] McGill M.R., Jaeschke H. (2019). Animal models of drug-induced liver injury. Biochim. Biophys. Acta (BBA) Mol. Basis Dis..

[13] Beger R.D., Bhattacharyya S., Yang X., Gill P.S., Schnackenberg L.K., Sun J., James L.P. (2015). Translational biomarkers of acetaminophen-induced acute liver injury. Arch. Toxicol..

[14] Gupta S., Stravitz R.T., Dent P., Hylemon P.B. (2001). Down-regulation of Cholesterol 7α-Hydroxylase (CYP7A1) Gene Expression by Bile Acids in Primary Rat Hepatocytes Is Mediated by the c-Jun N-terminal Kinase Pathway. J. Biol. Chem..

[15] Xie Y., Shen F., He Y., Guo C., Yang R., Cao H., Pan Q., Fan J. (2023). Gamma-Muricholic Acid Inhibits Nonalcoholic Steatohepatitis: Abolishment of Steatosis-Dependent Peroxidative Impairment by FXR/SHP/LXRα/FASN Signaling. Nutrients.

[16] Stolze L.F., Wendon J. (2014). Understanding Paracetamol-Induced Liver Failure. Intensive Care Med..

[17] Schadt H.S., Wolf A., Pognan F., Chibout S.-D., Merz M., Kullak-Ublick G.A. (2016). Bile acids in drug induced liver injury: Key players and surrogate markers. Clin. Res. Hepatol. Gastroenterol..

[18] Perez M.J., Briz O. (2009). Bile-Acid-Induced Cell Injury and Protection. World J. Gastroenterol..

[19] Zhang A., Sun H., Yan G., Han Y., Ye Y., Wang X. (2013). Urinary metabolic profiling identifies a key role for glycocholic acid in human liver cancer by ultra-performance liquid-chromatography coupled with high-definition mass spectrometry. Clin. Chim. Acta.

[20] Li Q., Chen F., Wang F. (2022). The immunological mechanisms and therapeutic potential in drug-induced liver injury: Lessons learned from acetaminophen hepatotoxicity. Cell Biosci..

[21] Ma F., Li L., Xu Z., Xie Y., Ma Y., Li P. (2025). Prognostic Modeling and Immune Infiltration Analysis in Hepatocellular Carcinoma Using Glutathione Metabolism-Associated Genes. Eur. J. Med. Res..

[22] Li H., Ye X., Hu Y., Wang Y., Ding Y., Yang Y., Mao R., Wu X., Dong H., Qiu K. (2025). Fibroblast growth factor receptor inhibitors ameliorate metabolic dysfunction-associated steatohepatitis by modulating the glycine-glutathione-gut microbiota axis. Free. Radic. Biol. Med..

[23] Muir K., Hazim A., He Y., Peyressatre M., Kim D.-Y., Song X., Beretta L. (2013). Proteomic and Lipidomic Signatures of Lipid Metabolism in NASH-Associated Hepatocellular Carcinoma. Cancer Res..

[24] Korbecki J., Bosiacki M., Kupnicka P., Barczak K., Ziętek P., Chlubek D., Baranowska-Bosiacka I. (2024). Biochemistry and Diseases Related to the Interconversion of Phosphatidylcholine, Phosphatidylethanolamine, and Phosphatidylserine. Int. J. Mol. Sci..

[25] Kanemaru K., Shimozawa M., Kitamata M., Furuishi R., Kayano H., Sukawa Y., Chiba Y., Fukuyama T., Hasegawa J., Nakanishi H. (2022). Plasma membrane phosphatidylinositol (4,5)-bisphosphate is critical for determination of epithelial characteristics. Nat. Commun..

[26] Kano K., Aoki J., Hla T. (2021). Lysophospholipid Mediators in Health and Disease. Annu. Rev. Pathol. Mech. Dis..

[27] Wen Y., Wang D., Wang H., Zhao J., Yao L., Zhang J., Chen B. (2025). A Glycerophospholipid Metabolism-Based Prognostic Model Guides Osteosarcoma Therapy. Transl. Oncol..

[28] Kur I.-M., Weigert A. (2024). Phosphatidylserine externalization as immune checkpoint in cancer. Pflügers Arch. Eur. J. Physiol..

[29] Lee J., Cheu J.W.-S., Wong C.C.-L. (2026). The Diverse Roles of Lipid Metabolism Reprogramming in Shaping the Tumor Immune Microenvironment. Cancer Res..

[30] Liang H., Song K. (2025). Multi-omics analysis reveals metabolic regulation of phosphatidylcholine, triglycerides, phosphatidylethanolamine, and cardiolipin metabolism in mouse liver with metabolic dysfunction-associated steatotic liver disease. PLoS ONE.

[31] Begriche K., Massart J., Robin M.-A., Borgne-Sanchez A., Fromenty B. (2011). Drug-induced toxicity on mitochondria and lipid metabolism: Mechanistic diversity and deleterious consequences for the liver. J. Hepatol..

[32] Liu J., Chen Y., Cen Z., Hong M., Zhang B., Luo X., Wang L., Li S., Xiao X., Long Q. (2025). Ganoderma lucidum spore oil attenuates acute liver injury by modulating lipid metabolism and gut microbiota. J. Pharm. Biomed. Anal..

[33] Rashid M., Varghese R.S., Ding Y., Ressom H.W. (2023). Biomarker Discovery for Hepatocellular Carcinoma in Patients with Liver Cirrhosis Using Untargeted Metabolomics and Lipidomics Studies. Metabolites.

[34] Tian J., Lu Y., Zhao Q.-L., Pu Q.-Y., Jiang S., Tang Y.-P. (2024). DHA-enriched phosphatidylserine alleviates bisphenol A-induced liver injury through regulating glycerophospholipid metabolism and the SIRT1-AMPK pathway. Heliyon.

[35] Atallah E., Freixo C., Alvarez-Alvarez I., Cubero F., Gerbes A.L., Kullak-Ublick G.A., Aithal G.P. (2021). Biomarkers of idiosyncratic drug-induced liver injury (DILI)—A systematic review. Expert Opin. Drug Metab. Toxicol..

[36] Jaber M.A., Ghanim B.Y., Al-Natour M., Abu Arqoub D., Abdallah Q., Abdelrazig S., Alkrad J.A., Kim D.-H., Qinna N.A. (2023). Potential biomarkers and metabolomics of acetaminophen-induced liver injury during alcohol consumption: A preclinical investigation on C57/BL6 mice. Toxicol. Appl. Pharmacol..

