# Supplementary material for: Widely Targeted Liver Metabolomics Reveals Potential Biomarkers in Mice with Drug-Induced Liver Injury
Source: Metabolites. 2026 Jan 28;16(2):96. doi: 10.3390/metabo16020096 (PMC12942010; doi:10.3390/metabo16020096)
Supplement: Supplementary file 1 [file metabolites-16-00096-s001.zip › metabolites-4100332-supplementary.pdf]

Supplementary material

Supplementary Figures

3.2. Stability and reliability Analysis of Detection Method

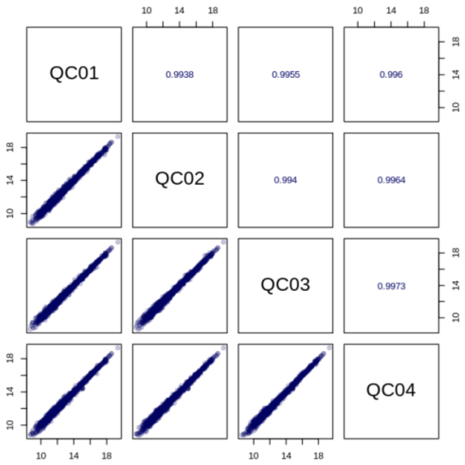

Figure S1. QC Sample Correlation Plot. The diagonal squares represent QC sample names. The lower-left squares display the corresponding QC sample correlation scatter plots, with metabolite content (log-transformed) as the x- and y-axes; each point represents a metabolite. The upper-right squares show the Pearson correlation coefficients for the corresponding QC samples.

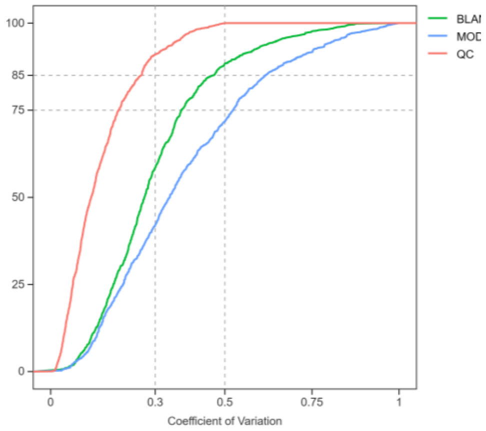

Figure S2. Empirical cumulative distribution function plot of the coefficient of variation (CV) values for QC samples.

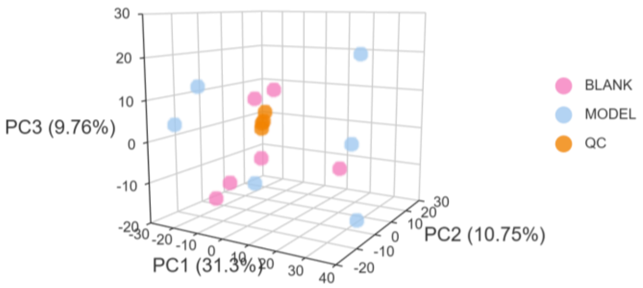

Figure S3. Principal Component Analysis Plot of Experimental Group Samples and QC Samples.

Supplementary Table

3.4. Widely-targeted Metabolomics Analysis of DILI Mice

Table S1. Mass spectrometric characterization of metabolites

Compounds	Q1 (Da)	Molecular weight (Da)	Ionization model	Formula
2-Methyllactic acid	103.04	104.1	[M-H]-	C4H8O3
(R)-2-Hydroxybutyric acid	103.04	104.1	[M-H]-	C4H8O3
Glu-Gln	274.1	275.1117	[M-H]-	C10H17N3O6
γ-Glu-Gln	274.1	275.111737	[M-H]-	C10H17N3O6
Indole-4-carboxaldehyde	144.05	145.0527639	[M-H]-	C9H7NO
Mucic acid	209.03	210.0375673	[M-H]-	C6H10O8
Glucaric acid	209.03	210.03757	[M-H]-	C6H10O8
Cholic acid	407.28	408.287575	[M-H]-	C24H40O5
γ-Muricholic acid	407.28	408.2876	[M-H]-	C24H40O5
L-Serine	106.05	105.043	[M+H]+	C3H7NO3
L-Isoserine	106.05	105.042594	[M+H]+	C3H7NO3
4-Methoxyestrone	301.18	300.172545	[M+H]+	C19H24O3
2-Methoxyestrone	301.18	300.172545	[M+H]+	C19H24O3
LPE(0:0/20:4)	500.28	501.285539	[M-H]-	C25H44NO7P
Acetylcholine	146.12	145.1102787	[M+H]+	C7H15NO2
4-Guanidinobutyric acid	146.09	145.085	[M+H]+	C5H11N3O2
LPC(16:2/0:0)	492.31	491.30119	[M+H]+	C24H46NO7P
LPC(0:0/16:2)	492.31	491.30119	[M+H]+	C24H46NO7P
Tauroursodeoxycholic acid	498.29	499.297	[M-H]-	C26H45NO6S
Hyodeoxycholic acid	391.29	392.293	[M-H]-	C24H40O4
13-HOTrE	293.21	294.1	[M-H]-	C18H30O3

D-Glucosamine 6-Phosphate	258.04	259.045706	[M-H]-	C6H14NO8P
9(S)-HpOTrE	309.21	310.2144094	[M-H]-	C18H30O4
3-Epideoxycholic acid	391.29	392.29266	[M-H]-	C24H40O4
Ribonic acid	165.04	166.13	[M-H]-	C5H10O6
2,2-Dimethylglutaric acid	159.07	160.17	[M-H]-	C7H12O4
Glycochenodeoxycholic acid 7-sulfate	528.26	529.270939	[M-H]-	C26H43NO8S
9-deoxy-9-methylene-PGE2	349.24	350.24571	[M-H]-	C21H34O4
Putrescine	89.11	88.1	[M+H]+	C4H12N2
Indole-3-Carboxaldehyde	146.06	145.053	[M+H]+	C9H7NO
Pterine	164.06	163.0494098	[M+H]+	C6H5N5O
SPH(d18:1)	300.29	299.282429	[M+H]+	C18H37NO2
3-Carboxypropyltrimethylammonium	146.12	145.110273	[M+H]+	C7H15NO2
Methionine Sulfoxide	166.05	165.045964	[M+H]+	C5H11NO3S
N-Methylisoleucine	146.12	145.110279	[M+H]+	C7H15NO2
1-Aminopentadecane	228.27	227.261299	[M+H]+	C15H33N
γ-Glu-Lys	276.16	275.14812	[M+H]+	C11H21N3O5
Met-Met	281.1	280.091533	[M+H]+	C10H20N2O3S2
3-Aminophenol	110.06	109.052764	[M+H]+	C6H7NO
γ-Glu-Phe	295.13	294.121573	[M+H]+	C14H18N2O5
Carnitine C6-OH	276.18	275.173274	[M+H]+	C13H25NO5
